# The Impact of Vitamin D Status on COVID-19 Severity among Hospitalized Patients in the Western Region of Saudi Arabia: A Retrospective Cross-Sectional Study

**DOI:** 10.3390/ijerph19031901

**Published:** 2022-02-08

**Authors:** Taqwa Bushnaq, Fadiyah Algethami, Alaa Qadhi, Reham Mustafa, Khloud Ghafouri, Wedad Azhar, Asma Al Malki

**Affiliations:** 1 Department of Food Science and Nutrition, College of Sciences, Taif University, P.O. Box 11099, Taif 21944, Saudi Arabia; asma@tu.edu.sa; 2 Clinical Nutrition Department, College of Applied Medical Sciences, University of Umm Al-Qura, P.O. Box 7067, Makkah 21955, Saudi Arabia; fadiyahalgethami2019@gmail.com (F.A.); ahqadhi@uqu.edu.sa (A.Q.); ramustafa@uqu.edu.sa (R.M.); Kjghafouri@uqu.edu.sa (K.G.); wfazhar@uqu.edu.sa (W.A.)

**Keywords:** coronavirus, COVID-19, vitamin D, infection and immunity

## Abstract

The coronaviruses disease 2019 (COVID-19) spreads continuously worldwide. The new vaccines and drugs have been approved. The prevention of disease is crucial, and some studies reveal the promising effect of alternative therapies such as vitamin D supplementations on COVID-19 prevention, but they still require sufficient evidence. Therefore, the current retrospective multicenter cross-sectional study aims to determine the primary association between the vitamin D status of hospitalized COVID-19 and its severity as well as mortality. A total of 197 COVID-19 were admitted at King Faisal Hospital, Al Noor Specialist Hospital in Makkah, and at Complex King Faisal Hospital in Taif in the Westering region of Saudi Arabia (SA) between June and August 2020. The demographic and clinical characteristics, laboratory tests included serum 25(OH)D and admission for intensive care unit (ICU), length of stay in the hospital, mechanical ventilation (MV) support, and mortality were recorded and analyzed. Vitamin D deficiency (25(OH)D < 20 ng/mL) was found in 73.10% of all study population. Multiple logistic regression was used after adjusted covariances such as age, gender, diabetes, hypertension, and chronic kidney disease (CKD). No statistically significant was shown for ICU admission [Odd Ratio, OR 1.25 (95% confidence interval, CI 0.41–3.88) *p* = 0.70], MV support [Odd Ratio, OR 3.12 (95% confidence interval, CI 0.74–13.21) *p =* 0.12] and mortality [Odd Ratio, OR 2.39 (95% confidence interval, CI 0.31–18.11), *p =* 0.40]. These data didn’t support the association between serum 25(OH)D and the severity of the disease among hospitalized COVID-19 patients.

## 1. Introduction

A novel coronavirus known as Acute Respiratory Syndrome Coronavirus 2 (SARS-CoV-2) was identified in Wuhan, China, at the end of 2019 [1,2]. COVID-19 was officially named for the disease caused by SARS-CoV-2 by the World Health Organization (WHO) on 11 February 2020 (WHO, 2020a) [3].The WHO classified COVID-19 as a global pandemic and a public health emergency on 11 March 2020 (WHO, 2020b) [4].

The clinical manifestations of COVID-19 vary from asymptomatic to life-threatening conditions [5]. The most common symptoms of COVID-19 are fever, fatigue, myalgia, and coughing. Less common symptoms are headache, runny nose, sputum production, sore throat, hemoptysis, and diarrhea [6]. Patients with severe symptoms require hospital admission and may develop complications such as acute respiratory distress syndrome (ARDS), acute respiratory injury, renal injury, arrhythmia, and septic shock, requiring intensive care unit (ICU) therapy [7,8].

Minimizing the rate of SARS-CoV-2 infection and the severity of COVID-19 symptoms is crucial to reducing the burden on the healthcare system. Therefore, several vaccines have been approved by WHO for COVID-19 prevention [9].

Vitamin D has several immunomodulation effects against viral infection [10]. It enhances the innate immune system by producing human antimicrobial peptides, such as cathelicidin and B-defensin, that lower the rate of viral infection [11].

However, vitamin D’s role in combating viral infection through macrophage defense is related to the cytokine response rather than killing the virus [12]. Moreover, it suppresses the cytokine storm by preventing the excessive production of pro-inflammatory cytokines [13]. Additionally, vitamin D modulates the adaptive immune system by suppressing the secretion of pro-inflammatory cytokines mediated by type-1 T helper cells. Moreover, they found testing for an elevated level of C-reactive protein (CRP), ferritin, and D-dimer “is a simple blood test that can help your healthcare provider determine if you may have a blood clotting condition” among all groups. IL6 was likely to be higher among the group with a severe vitamin D deficiency, but the result was not statistically significant. Additionally, they reported a mortality risk significantly higher among COVID-19 patients with vitamin D deficiency than in other groups (TNF), while it stimulates the production of anti-inflammatory cytokines mediated by type-2 T helper cells (Th2) [14,15]. Also, vitamin D is important in recruiting immune cells to infection sites and strengthening the junction integrity of epithelial cells (Grant et al., 2020). In addition, vitamin D regulates the renin-angiotensin system (RAS) by increasing the ratio of ACE2/ACE that leads to decreasing angiotensin 2 and enhancing the ACE2/Ang (1–7)/MasR axis. Consequently reducing the production of inflammatory cytokines and the risk of lung injury [16,17].

Several observational studies suggest a positive association between low serum(OH)D and the number of cases and mortality rate among hospitalized COVID-19 patients [18,19,20]. In contrast, a retrospective study in Newcastle in the UK demonstrated that serum 25(OH)D was not associated with mortality [21].

The scientific evidence related to the role of vitamin D in COVID-19 severity is currently limited in Arabian Gulf. To date, only one retrospective single center study conducted in Riyadh in Saudi Arabia (SA) reported no association between low serum 25(OH)D and infection of SARS-CoV-2, but mortality risk was high among COVID-19 with severe vitamin D deficiency [22]. Additionally, vitamin D deficiency is high among the Saudi population that may provide a unique direction in the association between vitamin D and COVID-19 [23].

Accordingly, this study investigates the impact of vitamin D status on the severity of hospitalized COVID-19 patients in terms of intensive care unit (ICU) admission, Mechanical Ventilation (MV) support, length of hospital stays, and mortality in the Western region of SA.

## 2. Materials and Methods

### 2.1. Study Design and Population

**Figure 1:**
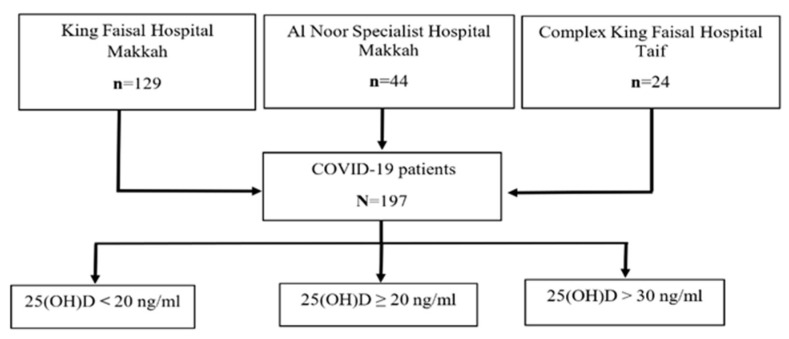
Algorithm for patient’s recruitment for study.

A retrospective, multicenter observational study was conducted in the Westering region of SA. Based on a statistical power of 80%, confidence level of 95%, and a margin of error of 5%, a minimum sample size of 197 COVID-19 was included. Both genders, aged 18 years and above, were recruited in the study from King Faisal Hospital in Makkah (N = 129), from Al Noor Specialist Hospital (N = 44) in Makkah, and from Complex King Faisal Hospital (N = 24) in Taif (Figure 1). Pregnant women, children, and patients with autoimmune diseases were excluded. The study was approved by the institutional review board (IRB) in the Taif region and given approval number 377.

### 2.2. Data Collection

The data of 197 COVID-19 patients were obtained from medical records between June to August 2020. Collected data included the hospital name and admission ward, gender, age, nationality, comorbidities, complications, length of stay in the hospital, as well as the severity of the disease depending on the lack of oxygen rate and Mechanical Ventilation (MV) support, intensive care unit) ICU (admission, and mortality.

### 2.3. Laboratory Measurements

The serum vitamin D level was measured for all COVID-19 patients during hospitalization by using the ADVIA Centaur XPT immunoassay system and vitamin D kit in King Faisal Hospital and Al Noor Specialist Hospital, and using the Cobos 6000 immunoassay with a Roch vitamin D kit in Complex King Faisal Hospital. Sensitivity should not be different, as observed earlier [24]. The vitamin D status for patients was determined based on their serum 25(OH)D levels, according to the local recommendations of diagnosis and treatment of vitamin D deficiency. The patients’ vitamin D status was classified as follows: adequate, 25(OH)D > 30 ng/mL; sufficient, 25(OH)D ≥ 20 ng/mL; and deficient, 25(OH)D < 20 ng/mL [25] (Figure 1).

Other laboratory parameters obtained from patient’s files include complete blood count (CBC), kidney function such as blood urea nitrogen (BUN) and creatinine, liver function such as alanine aminotransferase (ALA) and aspartate aminotransferase (AST), C-reactive protein (CRP), and serum vitamin D (25 OH).

### 2.4. Statistical Analysis

Data analysis was performed using the Statistical Package for the Social Sciences (SPSS) (IBM SPSS Statistics for Windows, Version 23.0. IBM Corp., Armonk, NY, USA). Frequency and percentages were used to display categorical variables, while means and standard deviation displayed continuous variables. Chi-squared test and Fisher’s exact test were used to test for the presence of an association between vitamin D level and categorical variables. The Shapiro–Wailk test and histogram chart were used to display data distribution. Square root logarithm equations were used to transfer normally distributed data. The Pearson correlation was used to assess the association between serum vitamin D and length of stay in hospital as well as serum CRP. A Post Hoc Test was used to determine the least significant difference (LSD) on the clinical outcome of COVID-19 patients based on serum vitamin D. Multivariable logistic regression was performed to determine the association between serum vitamin D and categorical variables with adjusting confounders such as age, gender and diabetes mellitus type 2 (DM), hypertension, and chronic kidney disease (CKD). The level of significance was set at 0.05.

## 3. Results

**Figure 2:**
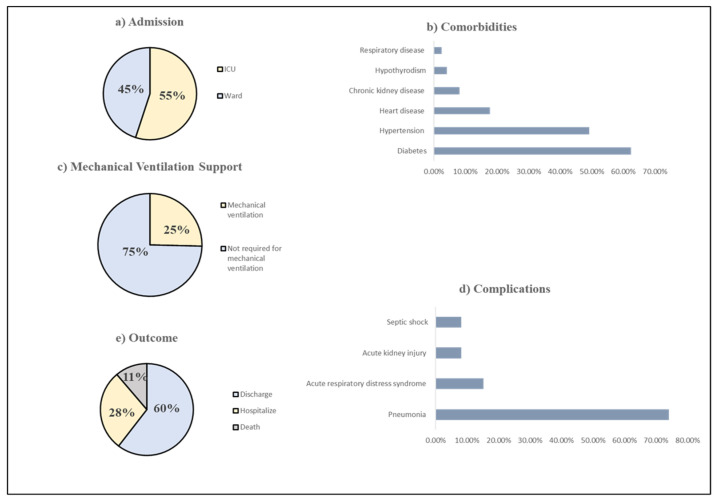
Clinical Characteristics and outcome of COVID 19 patients. (**a**) Admission; (**b**) Comorbidities; (**c**) Mechanical Ventilation Support (**d**) Complications (**e**) Outcome.

The baseline characteristics of COVID-19 patients are shown in Table 1. A total of 197 patients were included in the study. Among them, 47.72% were Saudi and 52.28% were non-Saudi. The vast majority of the patients were recruited from King Faisal Hospital in Makkah 129 (65.48%), followed by Al Noor Specialist Hospital in Makkah 44 (22.34%), and Complex King Faisal Hospital in Taif 24 (12.18%). A total of 109 (55%) patients were admitted to the general ward, while 88 (45%) patients were admitted to ICU (Figure 2). Patients were 67.51% male and 32.49% female. The mean age of patients was 57.26 years.

### 3.1. Clinical Features of COVID-19 Patients

As shown in Table 2, the most frequent comorbidities among patients were diabetes at 62.44%, followed by hypertension at 49.24%, cardiovascular disease at 17.77%, chronic kidney disease (CKD) at 8.12% while Hypothyroidism at 4.10%, and respiratory disease at 2.5% (Figure 2). The baseline biochemical markers are presented in Table 3. Most of the study population had a vitamin D deficiency with a mean serum of 25(OH)D 17.04 ± 11.18. The CRP was elevated among the study population with a mean of 17.15 ± 24.60. The creatinine and BUN levels were moderately increased with a mean of 133 ± 186.53 and 11.18 ± 13.44, respectively. The AST level was slightly affected with a mean of 59.83 ± 101.33.

### 3.2. The Association between Vitamin D Levels and the Severity of COVID-19

The association between vitamin D status and COVID-19 severity is presented in Table 4. The majority of the patients, 144 (73.10%), had 25(OH)D < 20 ng/mL while 33 (15.74%) patients had 25(OH)D ≥ 20 ng/mL, and only 22 (11.17%) patients had 25(OH)D > 30 ng/mL. No significant differences were found between COVID-19 patients admitted to the general ward or intensive care unit (ICU) (*p* = 0.67). Moreover, the main complications that patients developed during hospitalization were pneumonia 146 (74.11%), acute respiratory distress syndrome (ARDS) 30 (15.23%), acute kidney injury (AKI) 16 (8.12%), and septic shock 16 (18.12%) (Figure 2). However, no statistically significant differences in any one of these complications were found among patients in the three vitamin D groups. Additionally, 50 (25%) patients required mechanical ventilation (MV), which 147 (75%) patients did not require (Figure 2). Also, oxygen support was provided for 53 (26.90%) patients with 1–5 L/min, 20 (10.15%), 6–10 L/min, 30 (15.23%) patients with 11–15 L/min and 56 (28.43%) patients with >15 L/min or on MV, while 38 (19.29%) patients did not require oxygen support. No significant differences between MV or oxygen support were found among patients in the three vitamin D groups (*p* = 0.34, *p* = 0.49).

### 3.3. Clinical Outcome of COVID-19 Patients

The clinical outcome of COVID-19 patients is shown in Table 5. The mean length of stay in the hospital was 8.65 ± 0.52 days. There was no correlation between serum 25(OH)D and length of the hospital stay (r = 0.06, *p* = 0.41). Moreover, there is no association between serum 25(OH)D and CRP (r = −0.15. *p* = 11).

**Figure 3:**
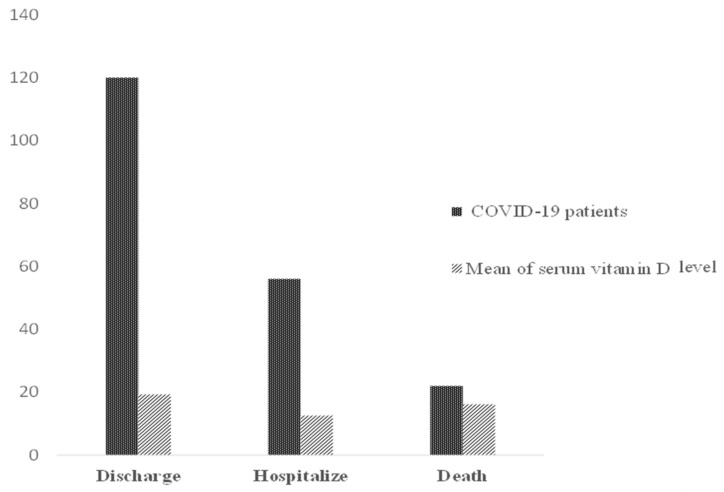
Number of COVID-19 patients with mean serum 25(OH)D.

In addition, 119 (60%) patients were discharged with mean serum 25(OH)D 18.98 ± 1.12; 22 (11%) of patients deceased with mean serum 25(OH)D 16.20 ± 2.41; while 56 (28%) patients were transferred to another hospital to receive inpatient care with mean serum 25(OH)D 13.23 ± 0.97. There was a significant difference in outcome of the COVID-19 patients (f = 3.81, *p* = 0.02) (Figure 3).

Table 6 shows the relationship of serum 25(OH)D level with clinical outcomes. There was no association between serum vitamin 25(OH)D and ICU admission (Odd Ratio, OR 1.25 [95% confidence interval, CI 0.41–3.88] *p* = 0.70), MV support (Odd Ratio, OR 3.12 [95% confidence interval, CI 0.74–13.21] *p =* 0.12) and mortality (Odd Ratio, OR 2.39 [95% confidence interval, CI 0.31–18.11] *p =* 0.40) in logistic regression after adjusted confounders.

However, in a logistic regression model containing serum 25(OH)D, diabetes was associated significantly with MV support with a 95% confidence interval, CI 1.16–5.67 *p* = 0.02. Age and diabetes were associated significantly with mortality with a 95% confidence interval (CI 0.92–0.98, *p* = 0.002) and 95% confidence interval (CI 1.05–15.76, *p =* 004), respectively.

## 4. Discussion

We investigated the hypothesis that vitamin D status is associated with COVID-19 severity. The objective of this study was to examine the association of vitamin D levels with admission to the intensive care unit (ICU), length of hospital stays, and clinical outcome of 197 hospitalized COVID-19 patients in the Western region of Saudi Arabia (SA). The most important clinically relevant finding in the current study indicated that vitamin D deficiency was highly prevalent among the study population at 73.10%. This result aligns with Alguwaihes et al. (2020), who performed a single center retrospective study involving 439 COVID-19 patients in the Riyadh region. They found that 74.7% of patients had a vitamin D deficiency, considered as one of the predictive factors of mortality [26]. This finding was expected as vitamin D deficiency is common in Saudi Arabia [23].

According to our analysis, the hospitalized and deceased patients had lower mean serum vitamin D than discharged patients. Low vitamin D levels have been associated with comorbidities [27,28], specifically in the elderly population (Kaur et al., 2019).

This might explain the current observations of this study, as it has been recently observed that more severe COVID-19 cases were common among the elderly with comorbidities [29]. The current study indicates that diabetes and age are the most significant predictors of the association between serum 25(OH)D and the mortality rate among COVID-19 patients. In contrast, Alguwaihes et al. (2020) demonstrated in a retrospective study conducted in Riyadh, SA, that diabetes is not associated with the mortality rate after control of a covariant among 439 COVID-19 [26].

However, it is noteworthy that in this study, vitamin D deficiency was not associated with mortality after adjustment for age, gender, and comorbidities such as type 2 DM, hypertension, and CKD. This agrees with a study conducted in the USA involving 93 COVID-19 patients. No association between vitamin D status and the mortality rate was found [30]. Moreover, this study did not find any association between serum vitamin D levels and adverse outcomes of COVID-19 patients and risk for ICU admission and MV support. These findings were confirmed in multi logistic regression after adjustment of covariates. The Szeto et al. (2020) results are aligned with the data from a retrospective case-control study that included 216 COVID-19 patients and 197 population-based control patients. They reported no association between serum vitamin D level and severity of COVID-19 symptoms, including ICU admission and intubation [31].

The present study’s findings do not support the previous research, which has suggested that vitamin deficiency was common among critically ill patients and associated with worse outcomes [32,33]. This inconsistency may be due to the difference in ethnic background, age group, and the modest sample size. On the other hand, Radujkovic et al. (2020) conducted a retrospective observational study among 185 COVID-19 patients in Germany. They demonstrated that patients with vitamin D deficiency had a 6 fold higher risk for developing a more severe course of the disease, including the requirement of invasive mechanical ventilation (IMV) and a 15 fold higher risk for death [34].

Furthermore, no association was detected in the current study between the serum vitamin D level and length of hospital stay in a multi-linear regression analysis. This result was also reported by Orchard et al. (2021), who performed a cohort observational study among 165 elderly patients and found that the serum vitamin D level was not associated with the number of days of hospitalization [35]. In contrast, Demir et al. (2021) demonstrated that COVID-19 patients with serum vitamin D > 30 ng/mL had a shorter hospital stay in a retrospective cohort study including 227 patients [36]. Possible explanations for this result may be the small sample size, that confounding factors were not controlled, and reliance on prehospitalization serum vitamin D values measured within six months before the diagnosis of COVID-19 rather than measuring during hospitalization.

These various studies indicate that vitamin D deficiency alone cannot fully explain the severity of COVID-19. A growing body of evidence supports the link between vitamin D supplementation and COVID-19 severity. A pilot randomized control trial involving 76 COVID-19 patients in Spain showed that 98% of the treated patients with calcifediol did not require ICU admission and did not die, while 50% of the untreated patients were admitted to the ICU and two died [37].

In addition, Annweiler et al. (2020) conducted a quasi-experimental study among 77 frail elderly COVID-19 patients and found that COVID-19 patients on regular vitamin D supplementation over the preceding year of 50,000 IU/month or 80,000 IU or 100,000 IU every 2–3 months had lower severity of COVID-19 (Odds Ratio (OR) = 0.08 (95% CI): 0.01; 0.81), *p* = 0.033) [38].

The absence of significant risk in COVID-19 severity among patients with hypovitaminosis D observed in the present study does not supersede the fact that vitamin D has an important role in supporting the immune system [39,40]. Thus, it is important to reach sufficient levels to avoid deficiency symptoms and comorbidities.

### Strength and Limitation

To the best of our knowledge, this retrospective study was the first multicenter study that included three hospitals in the western region of SA to assess the association between serum vitamin D level and severity of COVID-19. The categorization of vitamin D was based on local recommendations to diagnose vitamin D treatment and vitamin D deficiency. Multivariant regression was used to control the effect of possible confounders.

This study had several potential limitations as it was a retrospective study, and selection bias may be concerned. The sample size was small, the power calculation estimated at 80%. Some confounding factors such as dietary assessment, physical activity, ethnicity, smoking status, body mass index (BMI), and socioeconomic status weren’t considered for the patients. Vitamin D was measured for most hospitalized patients after COVID-19 had advanced to the acute phase rather than upon their first admission. The critical illness affected the vitamin D binding protein, which affects the vitamin D bioavailability [41]. Some patients underwent vitamin D supplementation during hospitalization, which wasn’t taken into consideration. Inflammatory markers such as CRP and ferritin weren’t available for most patients. Despite these limitations, the finding of this study added value to the literature, as evidence of COVID-19 patients in the middle east is limited and the results are mixed.

## 5. Conclusions

In conclusion, the prevalence of vitamin D deficiency is high among COVID-19 patients in the Western region of SA. The lack of association was detected in the present study between serum vitamin D and severity of COVID-19, including ICU admission, intubation, mortality, and days of hospitalization. Further large randomized control tail studies covering multiple institutions are needed to determine the therapeutic effect of vitamin D supplementation on COVID-19 severity.

## Figures and Tables

**Table 1:** Demographic characteristics of the study population.

Demographic Characteristic	N (%)
Hospital Admission King Faisal Hospital in Makkah Al-Noor Specialist Hospital in Makkah Complex King Faisal Hospital in Taif	129 (65.48%) 44 (22.34%) 24 (12.18%)
Ward Admission General Ward ICU	109 (55%) 88 (45%)
Nationality Saudi Non-Saudi	94 (47.72%) 103 (52.28%)
Gender Male Female	133 (67.51%) 64 (32.49%)
Age Minimum Maximum	57. 26 ± 15.74 ^a^ 20 97

^a^ Mean ± SD.

**Table 2:** Comorbidities of the study population.

Comorbidities	N (%)
Diabetes	123 (62.44%)
Hypertension	97 (49.24%)
Cardiovascular disease	35 (17.77%)
Chronic kidney disease	16 (8.12%)
Hypothyroidism	8 (4.10%)
Respiratory disease	5(2.53%)

**Table 3:** Biochemical analysis of the study population.

Parameters	Mean ± SD	Normal Range
25(OH)D	17.04 ± 11.18	30–70 ng/mL
CRP	17.15 ± 24.60	0–6 mg/L

Note: ng/mL = nanograms/millilitre, mg/L = milligrams per Liter, g/dL = grams per deciliter; umol/L = micromoles per liter, mmol/L = millimoles per litre, u/L = units per litre.

**Table 4:** Vitamin D according to the severity of COVID-19.

Clinical Outcome	Serum Vitamin D (ng/mL)				
	Deficiency (<20 ng/mL) N = 144 (73.10%)	Sufficient (≥20 ng/mL) N = 31 (15.74%)	Adequacy (>30 ng/mL) N = 22 (11.17%)		
**Ward Admission**				**N (%)**	***p*** **-Value**
General Ward	79	16	14	109 (55.33%)	0.67
ICU	65	15	8	88 (44.67%)	
**Complications**					
Pneumonia	104	24	18	146 (74.11%)	0.96
Acute respiratory distress syndrome	21	5	4	30 (15.23%)	0.85
Acute kidney injury	11	3	2	16 (8.12%)	0.76
Septic shock	12	2	2	16 (8.12%)	1.00
**Mechanical Ventilation**					
Mechanical ventilation support	40	7	3	50 (25%)	0.34
No mechanical ventilation support	104	24	19	147(75%)	
**Oxygen Support**					
No oxygen support	30	5	3	38 (19.29%)	0.49
1–5 L/min	37	7	9	53 (26.90%)	
6–10 L/min	13	4	3	20(10.15%)	
11–15 L/min	19	7	4	30 (15.23%)	
>15 L/min or on MV	45	8	3	56 (28. 43%)	

**Table 5:** Clinical outcome of COVID-19 patients based on serum vitamin D.

**Clinical Outcome**	**N (%)**	**Mean ± SD**	**R**	***p*** -Value *
Length of hospital stay	197 (100%)	8.65 ± 0.52	0.06	0.41
CRP	127 (64.5%)	17.14 ± 2.18	−0.15	0.11
**Clinical Outcome**	**N (%)**	**Serum Vitamin D (ng/mL)** **Mean ± SD**	**F**	***p*** -Value *
Discharge	119 (60%)	18.98 ± 1.12	3.81	0.02
Hospitalize (transfer)	56 (28%)	13.23 ± 0.97		
Deceased	22 (11%)	16.20 ± 2.41		

* Significant level *p* < 0.05.

**Table 6:** Multivariate logistic regression analysis for clinical outcome of COVID-19 patients.

Multiple Logistic Regression						
Clinical Outcome	ICU Admission		Mechanical Ventilation Support		Mortality	
Covariances	95% CI	*p*-Value	95% CI	*p*-Value	95% CI	*p*-Value *
Age	(0.97–1.00)	0.25	(0.96–1.00)	0.16	(0.92–0.98)	0.002
Gender	(0.96–3.46)	0.07	(0.62–2.75)	0.49	(0.31–2.45)	0.79
Diabetes	(0.71–2.54)	0.37	(1.16–5.67)	0.02	(1.05–15.76)	0.04
Hypertension	(0.37–1.39)	0.32	(0.36–1.61)	0.48	(0.16–1.36)	0.16
CKD	(0.49–4.08)	0.53	(0.37–3.84)	0.77	(0.63–1.00)	0.18
Adjusted ^a^ OR vit. D	1.25		3.12		2.39	
*p*–value *	0.70		0.12		0.40	
95% CI	(0.41–3.88)		(0.74–13.21)		(0.31–18.11)	

* Significant level *p* < 0.05; OR = Odds ratio; CI = confidence interval; ^a^ Logistic regression model containing vitamin D level as a continuous variable adjusted by age, gender, diabetes, hypertension, and chronic kidney disease (CKD).

## Data Availability

The data that support the findings of this study are available from [King Faisal Hospital, Al Noor Specialist Hospital, and from Complex King Faisal Hospital] but restrictions apply to the availability of these data, which were used under license for the current study, and so are not publicly available.
